# Mapping the Biotransformation of Coumarins through Filamentous Fungi

**DOI:** 10.3390/molecules24193531

**Published:** 2019-09-29

**Authors:** Jainara Santos do Nascimento, Wilson Elias Rozo Núñez, Valmore Henrique Pereira dos Santos, Josefina Aleu, Sílvio Cunha, Eliane de Oliveira Silva

**Affiliations:** 1 Organic Chemistry Department, Chemistry Institute, Federal University of Bahia, Salvador 40170-115, Bahia, Brazil; naranascimento14@gmail.com (J.S.d.N.); wrozo2502@yahoo.com (W.E.R.N.); hp20.2014@gmail.com (V.H.P.d.S.); silviodc@ufba.br (S.C.); 2 Organic Chemistry Department, Faculty of Sciences, University of Cádiz, 11510 Puerto Real, Cádiz, Spain; josefina.aleu@uca.es

**Keywords:** coumarin, biotransformation, filamentous fungi, selective hydroxylation

## Abstract

Natural coumarins are present in remarkable amounts as secondary metabolites in edible and medicinal plants, where they display interesting bioactivities. Considering the wide enzymatic arsenal of filamentous fungi, studies on the biotransformation of coumarins using these microorganisms have great importance in green chemical derivatization. Several reports on the biotransformation of coumarins using fungi have highlighted the achievement of chemical analogs with high selectivity by using mild and ecofriendly conditions. Prompted by the enormous pharmacological, alimentary, and chemical interest in coumarin-like compounds, this study evaluated the biotransformation of nine coumarin scaffolds using *Cunninghamella elegans* ATCC 10028b and *Aspergillus brasiliensis* ATCC 16404. The chemical reactions which were catalyzed by the microorganisms were highly selective. Among the nine studied coumarins, only two of them were biotransformed. One of the coumarins, 7-hydroxy-2,3-dihydrocyclopenta[*c*]chromen-4(1*H*)-one, was biotransformed into the new 7,9-dihydroxy-2,3-dihydrocyclopenta[*c*]chromen-4(1*H*)-one, which was generated by selective hydroxylation in an unactivated carbon. Our results highlight some chemical features of coumarin cores that are important to biotransformation using filamentous fungi.

## 1. Introduction

Natural heterocyclic products consisting of fused benzene and α-pyrone rings are designed as coumarins [1]. The great structural diversity of coumarins can be found both in simple coumarins, whose chemical structures contain only two rings, and in coumarins that contain an additional ring, such as furano- and pyranocoumarins. In all coumarin scaffolds, hydroxy or methoxy groups at position 7 are common structural features [2]. 

Natural coumarins are widespread in edible and medicinal plants as secondary metabolites [3]. These chemicals display broad biological activities [4], including antioxidant [5], antibacterial [6], antiviral [7], anti-inflammatory [8], antidepressant [9], and antitumoral activities [10], among others. In addition to other secondary metabolites, the biosynthesis of coumarins is controlled by several enzymes, and their accumulation is directly influenced by biotic and abiotic factors [11]. Given the wide pharmaceutical, chemical, and alimentary importance of coumarins, several alternative ways to achieve them have been developed in an attempt to replace the natural compound obtained by bioprospecting [12,13]. As a result, limitations associated with obtaining such substances in low yields from natural sources may be circumvented. 

Since the first synthetic coumarin [14], coumarin-like compounds have been mainly used in the pharmaceutical industry as precursors for the synthesis of anticoagulant drugs or in the production of fragrances. Several chemical compounds bearing a coumarin moiety continue to be produced by using synthetic methodologies [15,16], but some specific structural modifications and substituents insertions are barely performed. Sometimes, the synthetic methods require corrosive catalysts and long reaction times, and they generally generate byproducts along with the desired product [17]. 

Chemical derivatization using microorganisms (defined as biotransformation) represents a powerful tool for obtaining derivatives for fine chemical, pharmaceutical, and agrochemical industries according to green chemistry principles [18]. Biotransformation using microorganisms may operate in mild conditions (neutral pH, room temperature, and atmospheric pressure), and generally, it is conducive to high regio-, stereo-, and chemoselectivity at low cost [19]. Biotransformation processes have an interesting diversity, which allows for diverse products to be obtained from a single substrate [20]. Moreover, some chemical transformations that cannot be performed through the traditional synthetic methods are readily obtained using the biotransformation approach in ecofriendly reactions [21]. Within this context, the microbiological transformation of diterpenes has been studied to achieve the specific hydroxylation of unactivated C–H bonds, which is difficult to prepare using chemical methods [22]. 

Microbial biotransformation can be described as a reaction or a set of simultaneous reactions in which a precursor molecule is converted, rather than a fermentation process where molecules are produced from a carbon and energy source. Biotransformation could involve the use of enzymes or whole cells, or combinations thereof. In general, whole cells are more popular than isolated enzymes in industrial biotransformation [23] because the former allows for a great quantity of catalysts in small volumes and high turnover rates of enzymes and cofactors. Besides, filamentous fungi are especially useful as industrial enzyme sources because of their broad diversity in the production of proteins, which are quickly secreted [24]. 

Recently, our research group published a review on the biotransformation of simple, furano-, and pyranocoumarins using different genera of filamentous fungi [25]. The survey showed that *Cunninghamella* sp. and *Aspergillus* sp. are the most common genera used as catalysts in the biotransformation of several types of coumarins. These findings prompted us to investigate the chemical specificities of *Cunninghamella* and *Aspergillus* genera in transforming coumarin cores. 

Therefore, the present study reports on the biotransformation of a variety of coumarin compounds using *Cunninghamella elegans* ATCC 10028b and *Aspergillus brasiliensis* ATCC 16404. Our results provide an interesting way of mapping these microbial systems onto the derivatization of coumarin scaffolds. The approach used by us highlights some chemical features in the coumarin structures that allow for their biotransformation. Additionally, the biotransformation of coumarins using the selected fungi strains occurred under chemo- and stereoselectivity. One of the assays led to a selective hydroxylation at an unexpected position of the coumarin core.

## 2. Results and Discussion

Coumarins represent an important class of natural products and also synthetic oxygen-containing heterocycles. Several reports have claimed that the most common structural pattern in natural coumarins is hydroxylation at C-7. Based on this, the present study focused on an evaluation of the biotransformation of a panel of coumarins that contained one or two hydroxyl groups in the aromatic ring. 

**Figure 1:**
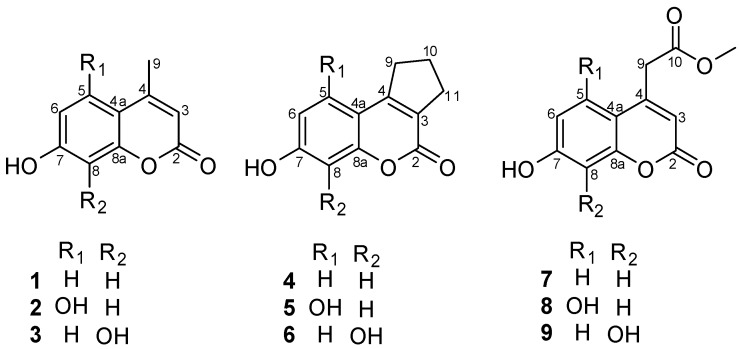
Chemical structures of coumarins used as substrates in biotransformation through *Cunninghamella elegans* ATCC 10028b and *Aspergillus brasiliensis* ATCC 16404.

Initial screening of the biotransformation of nine coumarin compounds (**1** to **9**, Figure 1) was carried out using the filamentous fungi *Cunninghamella elegans* ATCC 10028b and *Aspergillus brasiliensis* ATCC 16404. The biotransformations were carried out for 72 h, and samples were analyzed using HPLC every 24 h. Both fungi were able to transform only two of the coumarins (**4** and **7**) after 72 h of incubation. 

**Figure 2:**
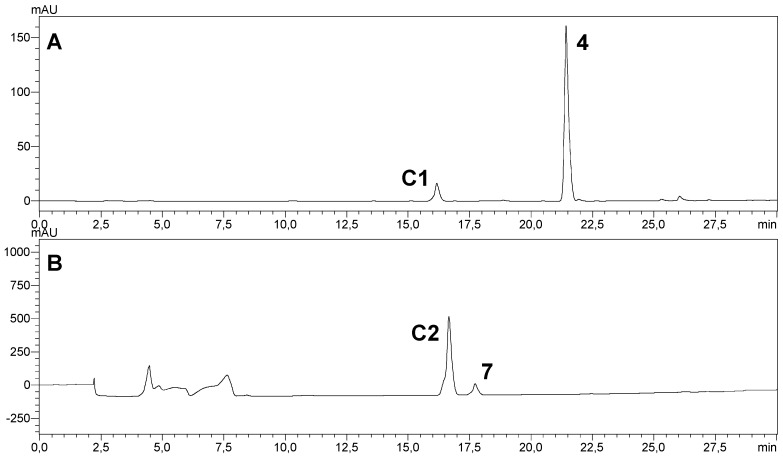
Reverse-phase HPLC elution profiles (λ = 211 nm) of the ethyl acetate extracts of *C. elegans* ATCC 10028b cultures incubated for 72 h with coumarin **4** (chromatogram A) and *A. brasiliensis* ATCC 16404 cultures incubated for 72 h with coumarin **7** (chromatogram B). The derivatives of coumarins **4** and **7** can be visualized in peaks **C1** and **C2**, respectively. AU: absorbance unit.

HPLC analysis (Figure 2) showed that *C. elegans* and *A. brasiliensis* biotransformed (exclusively) the coumarins **4** and **7** into more polar derivatives. The chemical structures of the coumarin substrates **4** and **7** contained two common features: aromatic rings from both contained only one hydroxyl group at C-7, and both coumarins contained a bulky group at C-4. None of the coumarins whose aromatic ring contained two hydroxyl groups (**2**, **3**, **5**, **6**, **8**, and **9**) were biotransformed by *C. elegans* or *A. brasiliensis*. Moreover, none of the coumarins with a methyl group at C-4 (**1**, **2**, and **3**) were biotransformed by the two evaluated filamentous fungi.

A more detailed analysis of the HPLC chemical profiles of the crude extracts of the biotransformations catalyzed by *C. elegans* and *A. brasiliensis* showed that both fungi biotransformed coumarins **4** and **7** into the same derivatives. However, the yields of derivatives **C1** and **C2** were different. Concerning the biotransformation of coumarin **4**, the yield of the assay catalyzed by *C. elegans* was five-fold higher than that catalyzed by *A. brasiliensis*. On the other hand, *A. brasiliensis* was more efficient in the biotransformation of coumarin **7**, generating almost double the yield. Similarly to our study, Lee and coworkers reported the biotransformation of isoflavone by *C. elegans* and *A. niger* [26]. The authors highlighted that *A. niger* gave a more complex metabolite profile than did *C. elegans*, but some derivatives were found in the crude extracts from both fungi. 

Next, the biotransformation experiments were repeated to facilitate the isolation and characterization of **C1** and **C2**. Scale-up biotransformations of coumarin-related compounds **4** and **7** by *C. elegans* and *A. brasiliensis*, respectively, led to the isolation of their main derivatives. 

**Figure 3:**
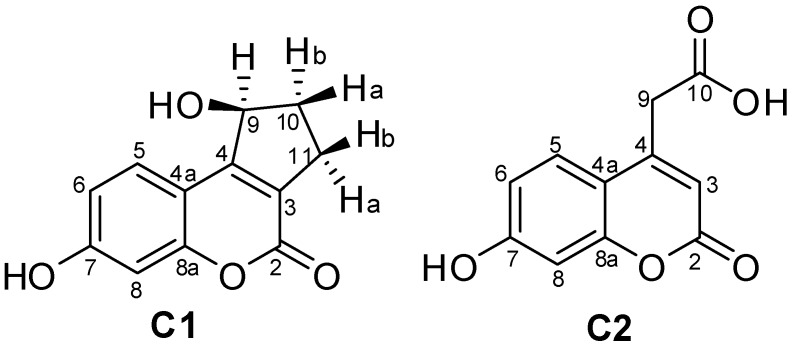
Chemical structures of **C1** and **C2**, which were isolated from the biotransformation of coumarins **4** and **7***,* respectively.

The derivative isolated from the biotransformation of coumarin **4** was identified as 7,9-dihydroxy-2,3-dihydrocyclopenta[*c*]chromen-4(1*H*)-one (**C1**), which is reported for the first time in the present study. Its molecular formula was determined as C_12_H_10_O_4_ by HRESIMS (*m/z* 217.0507 [M − H]^−^, Figure S6 in the Supplementary Materials), indicating that one new hydroxyl group was introduced into **4** as a result of the biotransformation. A comparison between the **4** and **C1** ^1^H NMR spectra (Figure S1 in the Supplementary Materials) showed that their chemical structures were very similar, except for C-9 (*δ* 75.1) in the **C1** chemical structure. The chemical shift of C-9 (*δ* 75.1) and the presence of only one hydrogen attached at C-9 (*δ* 5.20–5.22, ddd, H-9) indicated hydroxylation at this position. Heteronuclear HSQC and HMBC analysis, along with ^13^C NMR data analysis (see spectra in Figures S2–S4 in the Supplementary Materials), allowed for the unequivocal structural identification of **C1** (Figure 3). The substitution of one hydrogen for the hydroxyl group at C-9 explained the greater polarity of **C1** compared to its precursor, **4** (see chromatogram A, Figure 2). 

The hydroxylation at C-9 generated a chiral center in the **C1** chemical structure, which explained the multiplicity of the diastereotopic hydrogens attached at C-10 (*δ* 2.42–2.48, dddd, H-10a; and 2.00–2.05, dddd, H-10b) and C-11 (*δ* 3.17–3.23, dddd, H-11a; and 2.95–3.00 ddd, H-11b). The ddd *δ* 5.20–5.22 was attributed to the enantiotopic hydrogen attached at C-9. The optical rotation measurement of the **C1** sample ([α]^20^_D_ = −16°) showed that the biotransformation approach used herein was stereoselective with a preference for levogyre stereoisomer production. 

The magnitude of the *J* between two adjacent C–H bonds (^3^*J_HH_*) is directly dependent on the dihedral angle α between these two bonds [27]. Therefore, the *J* analysis of the ^1^H NMR signals of the hydrogens belonging to the **C1** cyclopentanol ring led to some conclusions about the relative stereochemistry of its substituents. The largest *J* value of ddd *δ* 5.20–5.22 (^3^*J_HH_* = 6.9 Hz) was attributed to the vicinal coupling between H-9 and H-10a (*δ* 2.42–2.48) that should occupy the opposite face of the cyclopentanol ring. By the same rationale, the middle *J* value (^3^*J_HH_* = 2.2 Hz) was attributed to the vicinal coupling between H-9 and H-10b (*δ* 2.00–2.05) that should occupy the same face of the cyclopentanol ring. Finally, the smallest *J* value of the ddd (^4^*J_HH_* = 1.4 Hz) was attributed to the W-coupling between H-9 and H-11a (*δ* 3.17–3.23). The W-coupling was herein confirmed through COSY homonuclear analysis (Figure S5 in the Supplementary Materials). 

Several coumarins and their derivatives have displayed interesting biological activities, and they are useful as a starting point for drug development [25]. The biotransformation approach employed in the present study provided chemo- and stereoselective hydroxylation of an unactivated C–H bond (position 9 of coumarin **4**). The ability of microorganisms to hydroxylate chemically inaccessible centers is a powerful synthetic tool because the functionalization of unactivated C–H bonds is a true challenge to organic synthesis [28]. Traditional chemical methods generally require highly reactive oxidizing agents, which causes difficulties in the regio- and stereocontrols. 

Considering the poor conversion rate of coumarin **4** and the consequent low yield of **C1** (9.0% yield), we investigated the biotransformation of **4** by *C. elegans*, intending to improve its conversion rate. The biotransformation of coumarin **4** by *C. elegans* was then carried out by using twice the amount of fungus and also by changing the incubation time to 120 and 168 h. We concluded that the biotransformation assay that used twice the amount of fungus for 72 h was the best one for the biotransformation of **4** into **C1**. Next, we repeated the biotransformation of **4** by *C. elegans* by using the optimized conditions, and we isolated **C1** with a yield of 22.0%. 

The next step of our study was the identification of the chemical structure of the derivative **C2**, which was achieved through biotransformation of the coumarin **7** by *A. brasiliensis* with a yield of 35.0%. The **C2** ^1^H NMR spectrum analysis (Figure S7 in the Supplementary Materials) showed that the biotransformation of **7** by *A. brasiliensis* led to hydrolysis at C-10. The ester group at C-10 of the chemical structure of **7** was converted into a carboxyl acid group in **C2**. The **C2** and **7** ^1^H NMR spectra were almost identical. Meanwhile, no methyl hydrogen was present in the **C2** ^1^H NMR spectrum. The ^1^H NMR of coumarin **7** contained a singlet at *δ* 3.83 with an integral value of 3. This signal was not found in the **C2** ^1^H NMR spectrum. All **C2** NMR data were in accord with those previously reported for 2-(7-hydroxy-2-oxo-2*H*-chromen-4-yl) acetic acid or 7-hydroxycoumarinyl-4-acetic acid [29]. 

It was previously established that C-7 substitution in the coumarin nucleus increases its antioxidant and antifungal activities [30]. Within this context, Molnar and coworkers have synthesized some coumarinyl thiosemicarbazides from **C2**. The synthesized compounds displayed interesting antibacterial activity against *Bacillus subtilis* [31]. 

As part of our studies on the biotransformation of coumarins, we recently reported that transformations at C-7, reductions at C3–C4, and lactone-ring opening were the most frequent reactions in coumarin cores submitted to biotransformation using filamentous fungi [25]. Unlike what was expected, the biotransformations described in the present study led to hydroxylation at an unexpected position and hydrolysis reaction. 

In summary, our investigations into the biotransformation of coumarin compounds by *C. elegans* and *A. brasiliensis* provided useful information about structural features that are important for the microbial transformation of this kind of compound. Coumarins that contained aromatic monohydroxylation and a bulky group at C-4 in their chemical structures were efficiently biotransformed by the fungi strains. Although both fungi strains converted the coumarins into the same derivatives, the reaction yields were distinct. We demonstrated the occurrence of a stereoselective hydroxylation at an unactivated carbon of one of the coumarins with a preference for levogyre stereoisomer production. The spectroscopic analysis allowed for the identification of a new chiral coumarin, whose relative stereochemistry was identified.

## 3. Materials and Methods 

### 3.1. General Analytical Procedures 

Nuclear magnetic resonance (NMR) spectra were recorded in CD_3_OD on a Varian VNMRS 600 (^1^H: 600 MHz; ^13^C: 150 MHz; Palo Alto, CA, USA) spectrometer operating at 25 °C or in DMSO-d_6_ on a Varian NMR AS 400 (^1^H: 400 MHz) spectrometer operating at 25 °C. The chemical shifts (*δ*) were assigned in ppm and the coupling constants (*J*) in Hz. The assignments were based on chemical shifts, integration, homonuclear (COSY), and heteronuclear (HMQC and HMBC) measurements. Optical rotation was measured at 20 °C in a Perkin Elmer 343 Polarimeter (Waltham, MA, USA) at 589 nm (sodium *D* line). Analytical HPLC analyses were carried out on a Shimadzu Shim-pack PREP-ODS(H)KIT 5 µm C_18_ column (4.6 × 250.0 mm, Kyoto, Japan), and the chemical profiles of the biotransformation and control extracts were obtained using 10% to 100% methanol in water containing 0.01% acetic acid over 30 min with a flow rate of 0.8 mL/min. The crude extracts were analyzed through the injection of 20 µL at 1 mg/mL on a Shimadzu (SIL-20A) multisolvent delivery system, a Shimadzu SPD-M20A, a photodiode array detector, and an Intel Celeron computer for analytical system control, data collection, and processing. The derivatives were isolated by using a chromatographic column (40 × 1.5 cm) containing silica gel (Sigma-Aldrich, 60 Å, Saint Louis, MO, USA). Mixtures of *n*-hexane (Synth) and ethyl acetate (Synth) were employed as a mobile phase. 

### 3.2. Substrates 

Nine synthetic coumarin analogs were submitted to the biotransformation experiments. All coumarins used as substrates were obtained according to known methods through a Pechmann reaction, and all of their spectroscopic data were identical to those previously described for 7-hydroxy-4-methyl-2*H*-2-chromenone (**1**) [32], 5,7-dihydroxy-4-methyl-2*H*-chromen-2-one (**2**) [33], 7,8-dihydroxy-4-methyl-2*H*-chromen-2-one (**3**) [34], 7-hydroxy-2,3-dihydrocyclopenta[*c*]chromen-4(1*H*)-one (**4**) [33], 7,9-dihydroxy-2,3-dihydrocyclopenta[*c*]chromen-4(1*H*)-one (**5**) [33], 6,7-dihydroxy-2,3-dihydrocyclopenta[*c*]chromen-4(1*H*)-one (**6**) [35], methyl 2-(7,8-dihydroxy-2-oxo-2*H*-chromen-4-yl)acetate (**7**) [36], methyl 2-(5,7-dihydroxy-2-oxo-2*H*-chromen-4-yl)acetate (**8**) [37], and methyl 2-(7-hydroxy-2-oxo-2*H*-chromen-4-yl)acetate (**9**) [38]. 

### 3.3. Biotransformation Assays 

The biotransformation of all coumarins (**1**–**9**) was done through screening with *Cunninghamella elegans* ATCC 10028b and *Aspergillus brasiliensis* ATCC 16404, which were obtained from the American Type Culture Collection (ATCC, Rockville, MD, USA). The filamentous fungi were maintained in 80% glycerol solution at −20 °C. 

The fungi were grown in a two-step culture procedure. First, each fungus was grown at 28 °C in Petri dishes containing malt agar (malt extract 2.0%, glucose 2.0%, peptone 0.1%, agar 1.8%) for 7 days. Next, an inoculum of five 6-mm disks containing mycelia and agar was added to 125-mL Erlenmeyer flasks, each holding 50 mL of Koch’s K1 medium (glucose 0.18%, peptone 0.06%, and yeast extract 0.04%). Coumarins (5 mg) were separately added to each flask as a solution in dimethyl sulfoxide (5 mg dissolved in 200 µL). Control flasks consisted of a culture medium with dimethyl sulfoxide and a fungus (without coumarin), a culture medium with coumarin and dimethyl sulfoxide (without a fungus), and a culture medium by itself. Biotransformation experiments were carried out at 28 °C for 72 h with shaking at 120 rpm. Samples were analyzed daily by HPLC. The mycelia were separated by filtration, the fermentation broths were extracted three times with ethyl acetate, and the solvent was evaporated under reduced pressure to yield crude extracts. All experiments were carried out in triplicate. 

Biotransformations of two selected coumarins (**4** and **7**) were separately carried out in 10 Erlenmeyer flasks (scale-up biotransformations) using the same aforementioned procedures. According to the yields of the biotransformation assays in the initial screening, the scale-up biotransformations of **4** and **7** were carried out by *C. elegans* and *A. brasiliensis*, respectively. The extraction of the culture broths by ethyl acetate was followed by evaporation of the solvent to yield the crude extracts of the biotransformations of **4** and **7** (41.0 mg and 59.0 mg, respectively). 

Additionally, the biotransformation conditions of coumarin **4** were investigated with a view toward increasing its conversion. For this, new assays were designed by using a greater quantity of the fungus *C. elegans* (10 6-mm disks containing mycelia and agar) and by increasing the incubation time (120 and 168 h). 

### 3.4. Isolation and Purification of the Derivatives 

The extracts from the culture broths of *C. elegans* or *A. brasiliensis* (scale-up biotransformations) with coumarins **4** and **7** were submitted to an isolation procedure (as described in Section 3.1) to yield the derivatives **C1** and **C2**, respectively. 

3′,7-dihydroxy-2,3-dihydrocyclopenta[*c*]chromen-4(1*H*)-one (**C1**): 3.6 mg (9.0% yield) brown powder; [α]^20^_D_ =−16° (0.34; CH_3_OH); ^1^H NMR (600 MHz, CD_3_OD) 7.44 (d, *J* = 8.4 Hz, 1H, H-5), 6.77 (dd, *J* = 8.4 and 2.2 Hz, 1H, H-6), 6.68 (d, *J* = 2.2 Hz, 1H, H-8), 5.20–5.22 (ddd, *J* = 6.9, 2.2, and 1.4 Hz, 1H, H-9), 3.17–3.23 (dddd, *J* = 18.0, 8.0, 6.9, and 1.4 Hz, 1H, H-11a), 2.95–3.00 (ddd, *J* = 18.0, 9.1, and 3.3 Hz, 1H, H-11b), 2.42–2.48 (dddd, *J* = 13.5, 9.1, 6.9, and 6.9 Hz, 1H, H-10a), 2.00–2.05 (dddd, *J* = 13.5, 8.0, 3.3, and 2.2 Hz, 1H, H-10b); ^13^C NMR (150 MHz, CD_3_OD) 163.1 (C-2), 162.2 (C-7), 161.3 (C-4), 158.2 (C-8a), 127.8 (C-5 and C-3), 115.5 (C-6), 103.8 (C-4a and C-8), 75.1 (C-9), 34.2 (C-10), 30.3 (C-11). HRESIMS *m/z* 217.0507 [M -H]^−^ (calcd for [M −H]^−^ 217.0501). 

2-(7-hydroxy-2-oxo-2*H*-chromen-4-yl) acetic acid (**C2**): 13.0 mg (35.0% yield) brown powder; ^1^H NMR (400 MHz, DMSO-d_6_) 12.75 (br s, 1H, COOH), 10.57 (s, 1H, OH-7), 7.54 (d, *J* = 8.7 Hz, 1H, H-5), 6.82 (dd, *J* = 8.7 and 2.3 Hz, 1H, H-6), 6.74 (d, *J* = 2.3 Hz, 1H, H-8), 6.23 (s, 1H, H-3), 3.83 (s, 2H, H-9).

## Supplementary Materials

The following are available online, Figure S1: ^1^H NMR spectrum of compound **C1** (600 MHz, CD_3_OD); Figure S2: ^13^C NMR spectrum of compound **C1** (150 MHz, CD_3_OD); Figure S3: ^1^H-^13^C HSQC 2D NMR correlation spectroscopy of compound **C1** (600 MHz/150 MHz, CD_3_OD); Figure S4: ^1^H-^13^C HMBC 2D NMR correlation spectroscopy of compound **C1** (600 MHz/150 MHz, CD_3_OD); Figure S5: ^1^H-^1^H COSY 2D NMR correlation spectroscopy of compound **C1** (600 MHz, CD_3_OD); Figure S6: HRESIMS spectrum of compound **C1** (negative ion mode); Figure S7: ^1^H NMR spectrum of compound **C2** (400 MHz, DMSO-d_6_).
