# Supplementary material for: Mapping the Biotransformation of Coumarins through Filamentous Fungi
Source: Molecules. 2019 Sep 29;24(19):3531. doi: 10.3390/molecules24193531 (PMC6803992; doi:10.3390/molecules24193531)
Supplement: Supplementary file 1 [file molecules-24-03531-s001.pdf]

Supplementary data

Mapping the biotransformation of coumarins by filamentous fungi

Jainara Santos do Nascimento[1], Wilson Elias Rozo Núñez[1], Valmore Henrique Pereira dos Santos[1], Josefina Aleu[2], Sílvio Cunha[1], Eliane de Oliveira Silva[1,*]

[1]Organic Chemistry Department, Chemistry Institute, Federal University of Bahia, 40170-115 Salvador, Bahia, Brazil

[2]Organic Chemistry Department, Faculty of Sciences, University of Cádiz, 11510 Puerto Real, Cádiz, Spain

*Corresponding author at: Organic Chemistry Department, Chemistry Institute, Federal University of Bahia, Barão de Jeremoabo 147, Salvador 40170-115, Bahia, Brazil.

E-mail address: elianeos@ufba.br Telephone number: +55 71 32836893

ORCID number: 0000-0001-9121-0481

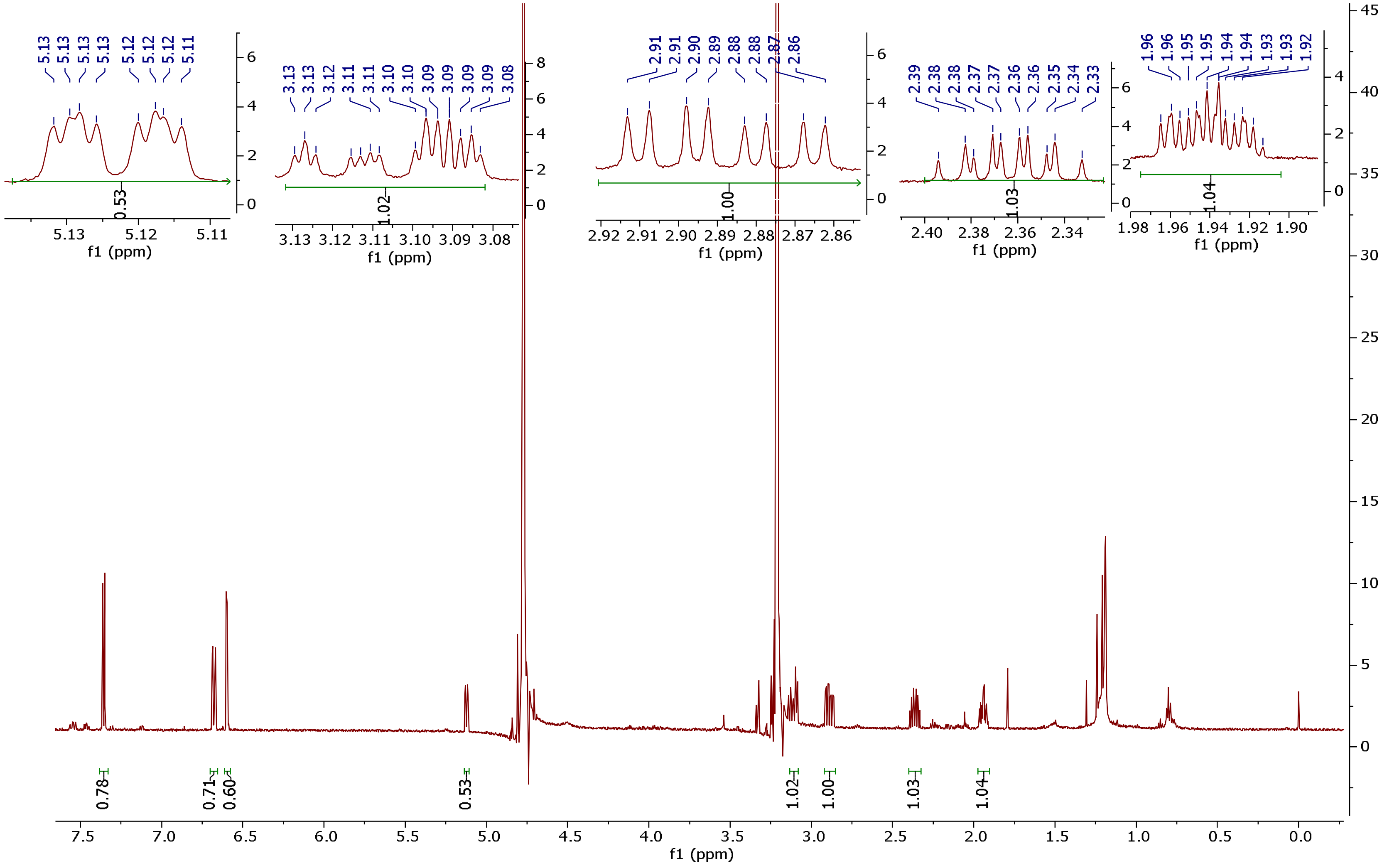

Figure S1: ^{1}H NMR spectrum of compound **C1** (600 MHz, CD_3OD)

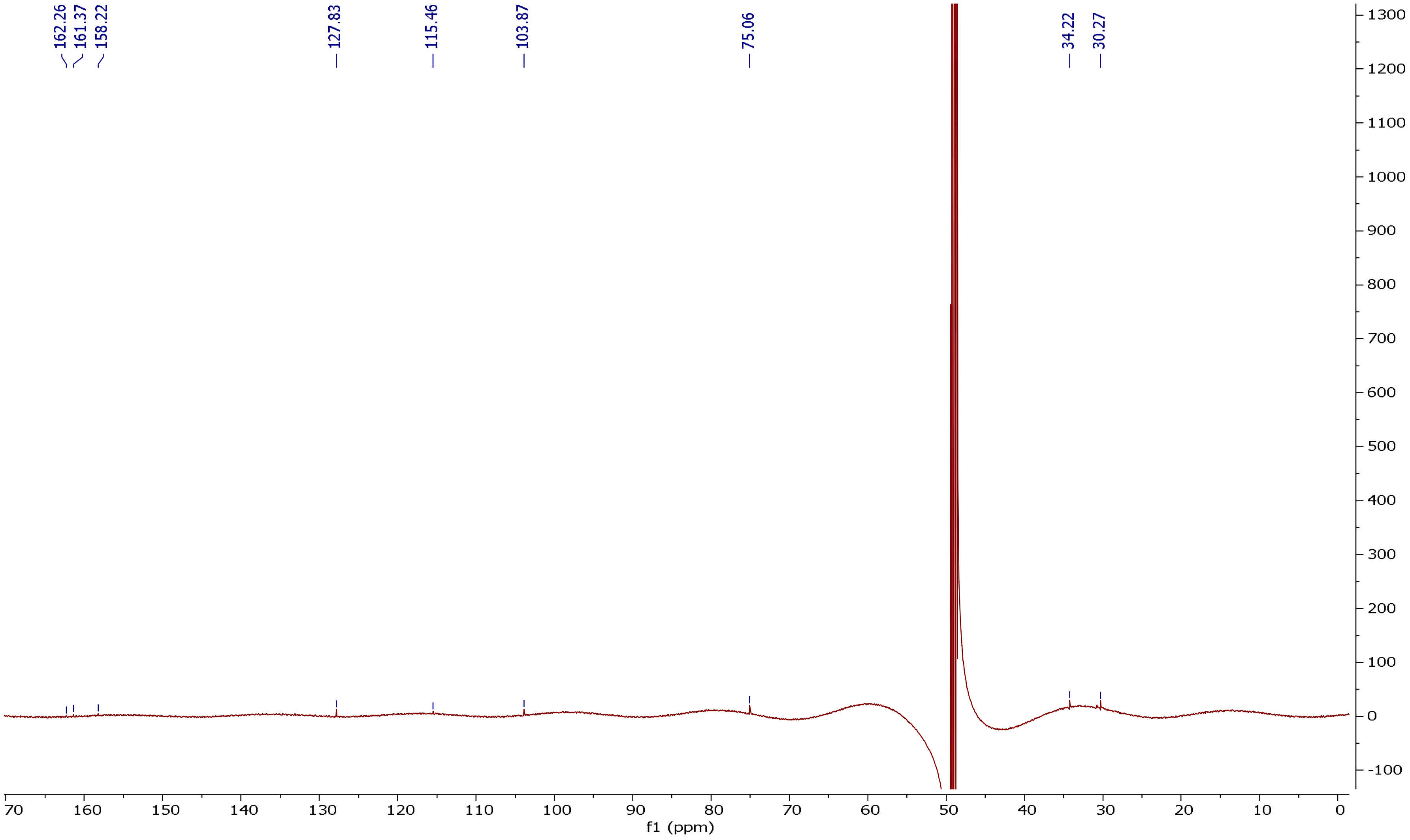

Figure S2: ^{13}C NMR spectrum of compound **C1** (150 MHz, CD_3OD)

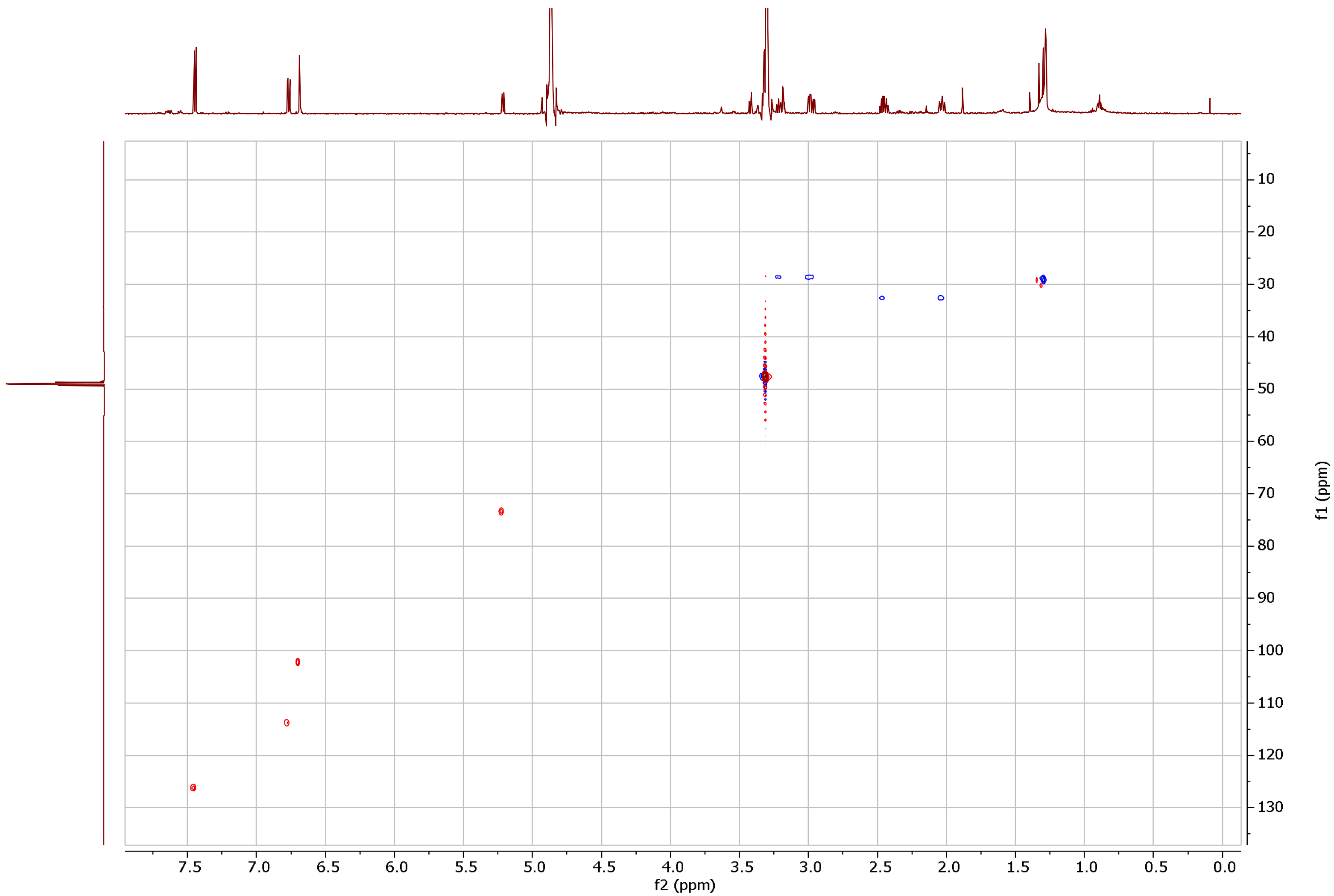

Figure S3: ^{1}H-^{13}C HSQC 2D NMR correlation spectroscopy of compound **C1** (600 MHz/150 MHz, CD_3OD)

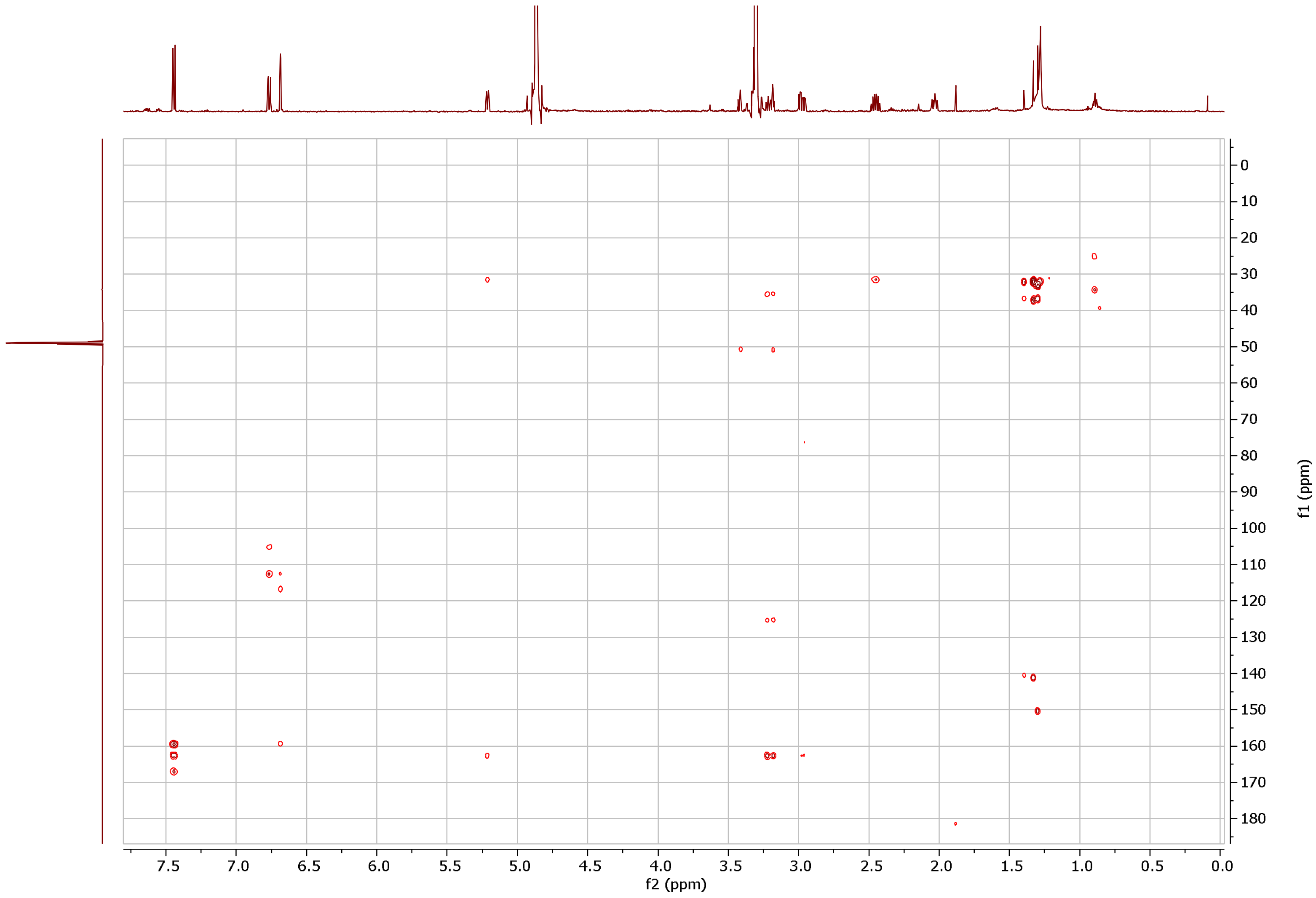

Figure S4: ^{1}H-^{13}C HMBC 2D NMR correlation spectroscopy of compound **C1** (600 MHz/150 MHz, CD_3OD)

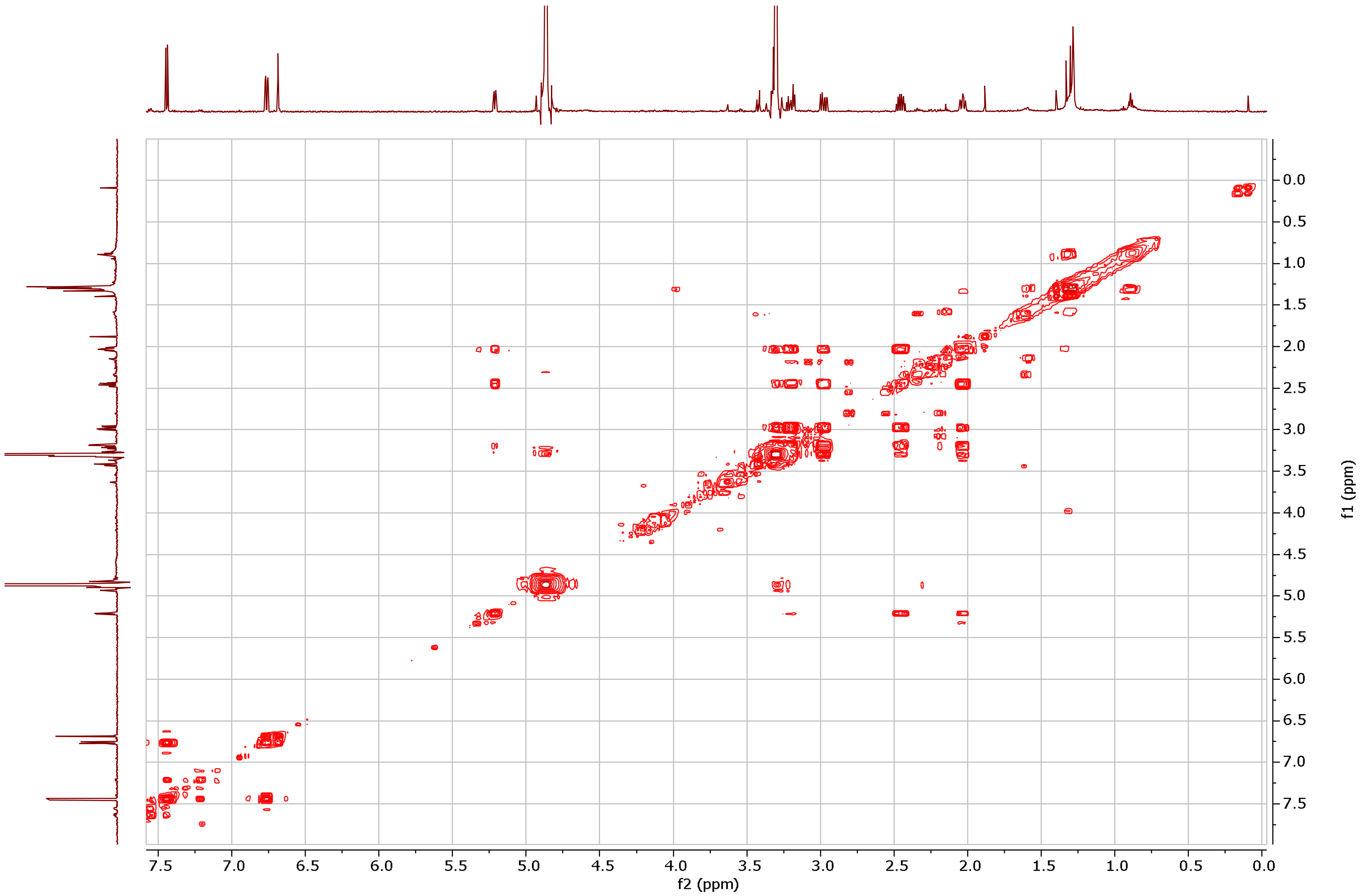

Figure S5: ^{1}H-^{1}H COSY 2D NMR correlation spectroscopy of compound **C1** (600 MHz, CD_3OD)

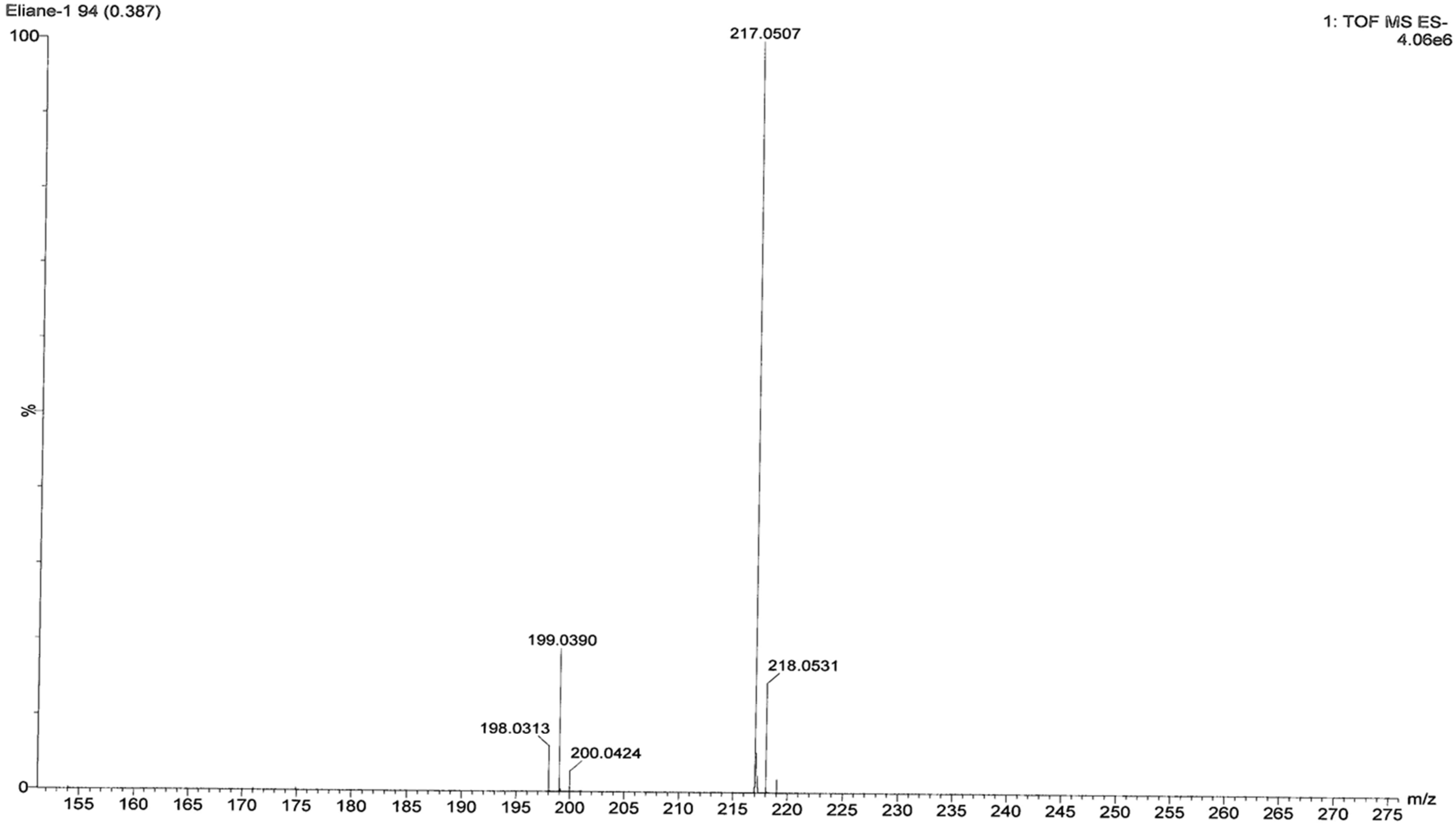

Figure S6: HRESIMS spectrum of compound **C1** (negative ion mode)

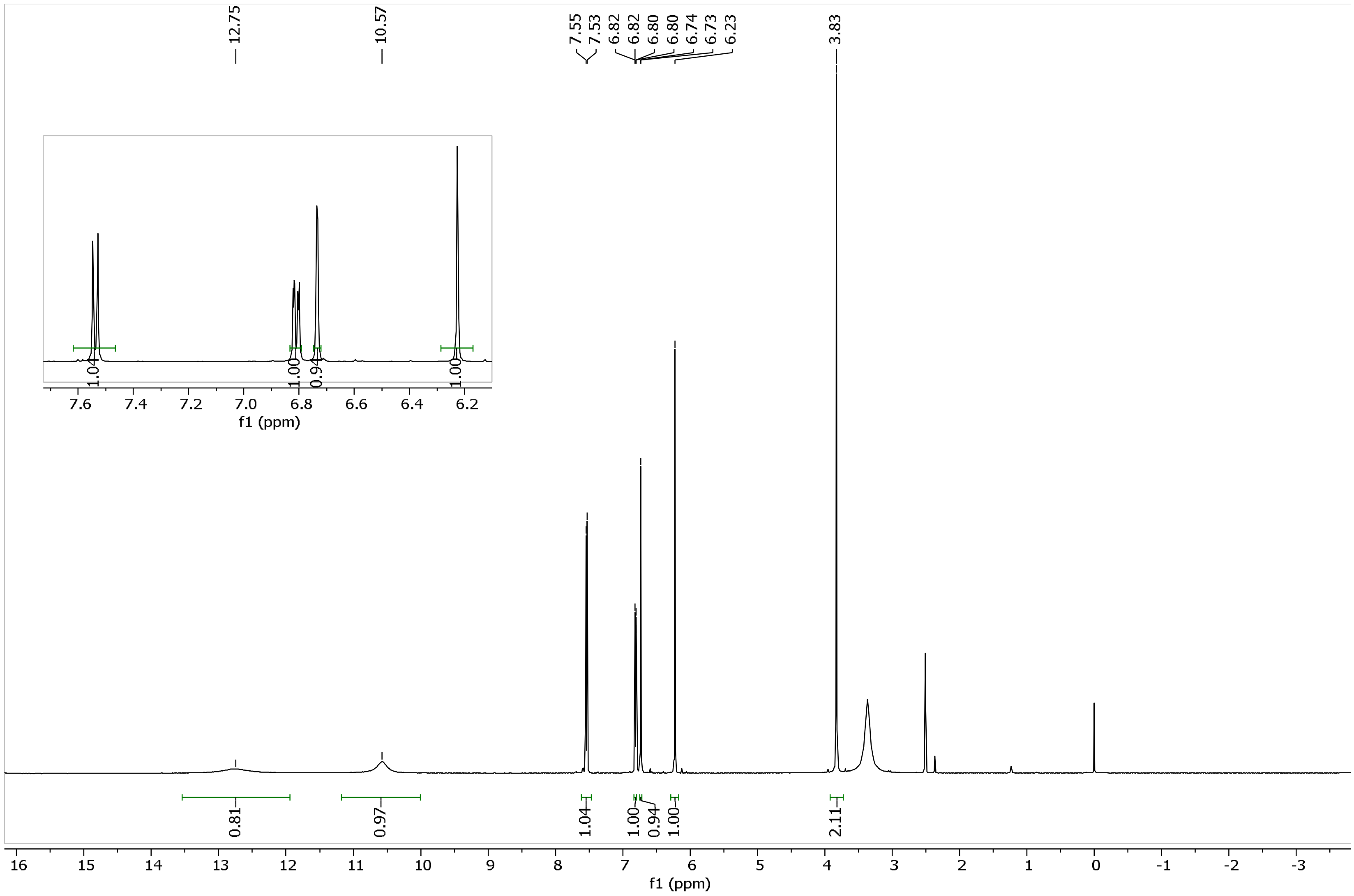

Figure S7: ^{1}H NMR spectrum of compound **C2** (400 MHz, DMSO-d$_6$)
